# Seasonal Influenza Split Vaccines Confer Partial Cross-Protection against Heterologous Influenza Virus in Ferrets When Combined with the CAF01 Adjuvant

**DOI:** 10.3389/fimmu.2017.01928

**Published:** 2018-01-08

**Authors:** Dennis Christensen, Jan P. Christensen, Karen S. Korsholm, Louise K. Isling, Karin Erneholm, Allan R. Thomsen, Peter Andersen

**Affiliations:** ^1^Department of Infectious Disease Immunology, Division of Vaccine, Statens Serum Institut, Copenhagen, Denmark; ^2^Department of Immunology and Microbiology, University of Copenhagen, Copenhagen, Denmark; ^3^Department of Quality Assurance/Quality Control, Section of Biological Service, Division of Vaccine, Statens Serum Institut, Copenhagen, Denmark

**Keywords:** influenza A virus, adjuvants, immunologic, heterologous immunity, ferrets, T cells

## Abstract

Influenza epidemics occur annually, and estimated 5–10% of the adult population and 20–30% of children will become ill from influenza infection. Seasonal vaccines primarily work through the induction of neutralizing antibodies against the principal surface antigen hemagglutinin (HA). This important role of HA-specific antibodies explains why previous pandemics have emerged when new HAs have appeared in circulating human viruses. It has long been recognized that influenza virus-specific CD4(+) T cells are important in protection from infection through direct effector mechanisms or by providing help to B cells and CD8(+) T cells. However, the seasonal influenza vaccine is poor at inducing CD4(+) T-cell responses and needs to be combined with an adjuvant facilitating this response. In this study, we applied the ferret model to investigate the cross-protective efficacy of a heterologous trivalent influenza split-virion (TIV) vaccine adjuvanted with the CAF01 adjuvant, with proven ability to induce CD4(+) T-cell and antibody responses in mice, ferrets, pigs, primates, and humans. Our results indicate that CAF01-adjuvanted vaccine induces HA inhibition (HAI)-independent protection after heterologous challenge, manifested as reduced viral load and fever. On the other hand, we observe increased inflammation in the airways and more neutrophil and mononuclear cell infiltration in these ferrets when compared with optimally protected animals, i.e., ferrets receiving the same vaccine but a homologous challenge. This suggest that HAI-independent immunity induced by TIV + CAF01 can reduce viral shedding and systemic disease symptoms, but does not reduce local inflammation in the nasal cavity.

## Introduction

Annual influenza epidemics cause illness in up to 30% of the population dependent on age and general health status. Worldwide, these annual epidemics are estimated to result in 3–5 million cases of severe illness, and about 250,000–500,000 deaths, especially among children, elderly, and immune-deprived individuals (http://www.who.int/mediacentre/factsheets/fs211/en/).

In addition to this, new strains occasionally appear, reflecting mutations and recent re-assortments, causing potentially life-threatening pandemics. In the past century, four such pandemics have occurred with the most lethal being the Spanish flu (1918–1920) killing between 50 and 100 million people, making it one of the most deadly disease outbreaks in history (1). For the past 20 years, there have been a number of highly pathogenic avian influenza strains circulating, which occasionally have crossed over to humans, in some cases with fatal outcome as recently reported for avian H10N8 (2), H7N9 (3), and H5N6 (4). Fortunately, human-to-human transmission of such highly lethal strains have not yet been reported, but it may only be a question of time before a human adapted variant will appear.

Seasonal flu vaccines primarily work through the induction of neutralizing antibodies against the principal surface antigen hemagglutinin (HA). HA attaches to host cell sialic acid receptors thus mediating infection of the cell. Neutralizing antibodies directed toward HA therefore reduce infection and they can even confer sterilizing immunity. This important role of HA-specific antibodies explains why previous pandemics have emerged when new HAs have appeared in viruses circulating in the human population. The latest pandemic in 2009 thus emerged when the circulating swine lineage of H1 HA, replaced the circulating human lineage (A/Brisbane/59/2007). The emerging H1N1 strain (A/California/04/2009) diverged from recent H1 variants with up to 50% in the residues corresponding to the antigenic sites of HA and were more closely related to the H1 HA strain causing the 1918 pandemic, with as little as 20% residue difference (5). To prevent or reduce severity of future influenza pandemics, it is therefore important to develop influenza vaccines, which do not rely so heavily on HA neutralization.

Cell-mediated immune responses against highly conserved epitopes in proteins such as the matrix proteins (M1 and M2), the nucleoprotein, and the polymerase basic proteins (PB1 and PB2) have been shown to mediate heterologous and heterosubtypic immunity against influenza A (6, 7). Cytotoxic CD8+ T cells are thought to be the primary mediators, but there is mounting evidence that also CD4+ T cells can facilitate of heterologous/-subtypic immunity although the underlying mechanisms are still not completely clear (8, 9). The main view is that CD4+ T cells provide help to CD8+T cells and B cells to promote viral clearance (8, 10). It has, however, been shown that both TH1 and TH17 cells transferred into naive mice can protect them against influenza, whereas TH2 cells do not play a role (11). This suggests that induction of TH1 and TH17 responses to the seasonal trivalent influenza split-virion (TIV) vaccine may increase cross-protection against heterologous/-heterosubtypic influenza strains and that the combination of TIV with the right adjuvant may facilitate this.

Several novel candidate adjuvants in clinical development have been assessed within the Advanced Immunization Technologies collaborative research program. Among these, the CAF01 adjuvant was recently shown to generate the strongest T-cell response in mice and that this T-cell response was biased toward a mixed TH1/TH17 response (12–15). This strong T-cell induction has been obtained in both mice, ferrets, pigs, non-human primates, calves, and humans (12, 16–21).

In this study, we applied the ferret model to investigate the cross-protective efficacy of T cells generated by a heterologous TIV vaccine adjuvanted with CAF01. The ferret model has for long been the preferred model for testing human influenza vaccines, the main reason being that ferrets share host range, clinical symptoms, and pathological changes in the respiratory tract with humans (22). On the other hand, the ferret model is difficult with respect to monitoring immune responses. The serum antibody responses to influenza infection and vaccination are much similar in humans and ferrets, and the methods are well established. However, the ferret model has only few tools for monitoring innate and adaptive cell-mediated immune responses. Vaccine efficacy determined in the ferret model therefore has to rely on monitoring of clinical symptoms and humoral responses. This complicates the evaluation of the cross protectiveness of influenza vaccines in situations where HA neutralization does not play a critical role. We therefore combine the classical clinical monitoring with immunohistological examination of the infection site to study the cross-protective efficacy of CAF01-adjuvanted TIV.

## Results

Our primary goal was to evaluate whether CAF01 could enhance the heterologous protection by a TIV vaccine. Thus, we immunized four groups of ferrets with adjuvanted or non-adjuvanted suboptimal doses (1/10 human dose) of TIV vaccines containing split-virion proteins from Influenza B, H3N2, and H1N1 of either the A/Brisbane/59/2007 or the A/California/04/2009 strains (designated TIV2007 and TIV2009, respectively) (Table 1). Unimmunized ferrets were used as controls.

**Table 1:** Vaccination groups.

Vaccine strain compared to challenge virus	Vaccine		Challenge virus strain
	Without adjuvant	With adjuvant	
Homologous	Trivalent influenza split-virion (TIV)2007	TIV2007 + CAF01	A/Brisbane/59/2007
Heterologous	TIV2009	TIV2009 + CAF01	

The ferrets were immunized twice 3 weeks apart. All groups including the unimmunized control group were challenged intranasally 4 weeks after last immunization, with 10^5^ TCID50 of the A/Brisbane/59/2007 H1N1 influenza strain. Thus, two vaccination groups were challenged with a homologous virus and the other two groups were challenged with a heterologous virus (Table 1). We also tested the reverse combinations using A/California/04/2009 as challenge strain. These data are not shown since the results were equivalent.

### Viral Load

**Figure 1:**
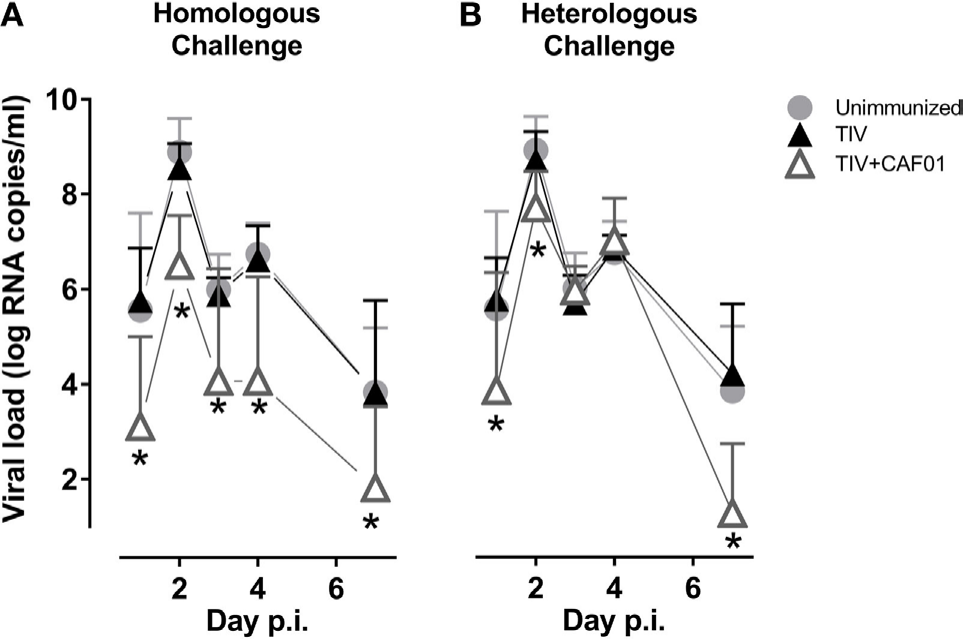
CAF01 enhances the protection of a split flu vaccine both against a homologous and a heterologous challenge. Ferrets immunized two times with either the seasonal trivalent influenza split-virion (TIV)2007 **(A)** or the seasonal TIV2009 **(B)** vaccine without adjuvant (▴) or with CAF01 adjuvant (▵). Unimmunized controls (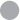). Challenge with the A/Brisbane/59/2007 influenza strain. Viral load in nasopharyngeal swab samples was determined by quantitative real-time polymerase chain reaction. Shown are mean values + SD (*n* = 8). Statistical analysis: repeated-measures two-way ANOVA based on the data shown in each graph. Dunnet’s test was used to compare unimmunized to the two other groups. Asterisks show statistically significant differences compared to the unimmunized group.

The ability of the vaccines to inhibit influenza infection was investigated by swabbing the nasal cavity of the ferrets from 1 to 7 days after challenge and determining the viral load by quantitative RT-PCR (Figure 1). The ferrets immunized with the non-adjuvanted vaccines were not protected at the chosen suboptimal dose of homologous and heterologous TIV. By contrast, the CAF01-adjuvanted TIV vaccine significantly reduced the viral load at all time points after homologous challenge. In addition, the CAF01-adjuvanted vaccine reduced the viral load after heterologous challenge at both the two earliest and at the latest time points investigated, showing that adjuvanting the TIV with CAF01 increases the resistance to challenge with a heterologous influenza virus. The reduction in viral load was less pronounced after heterologous challenge.

### Humoral Immunity

**Figure 2:**
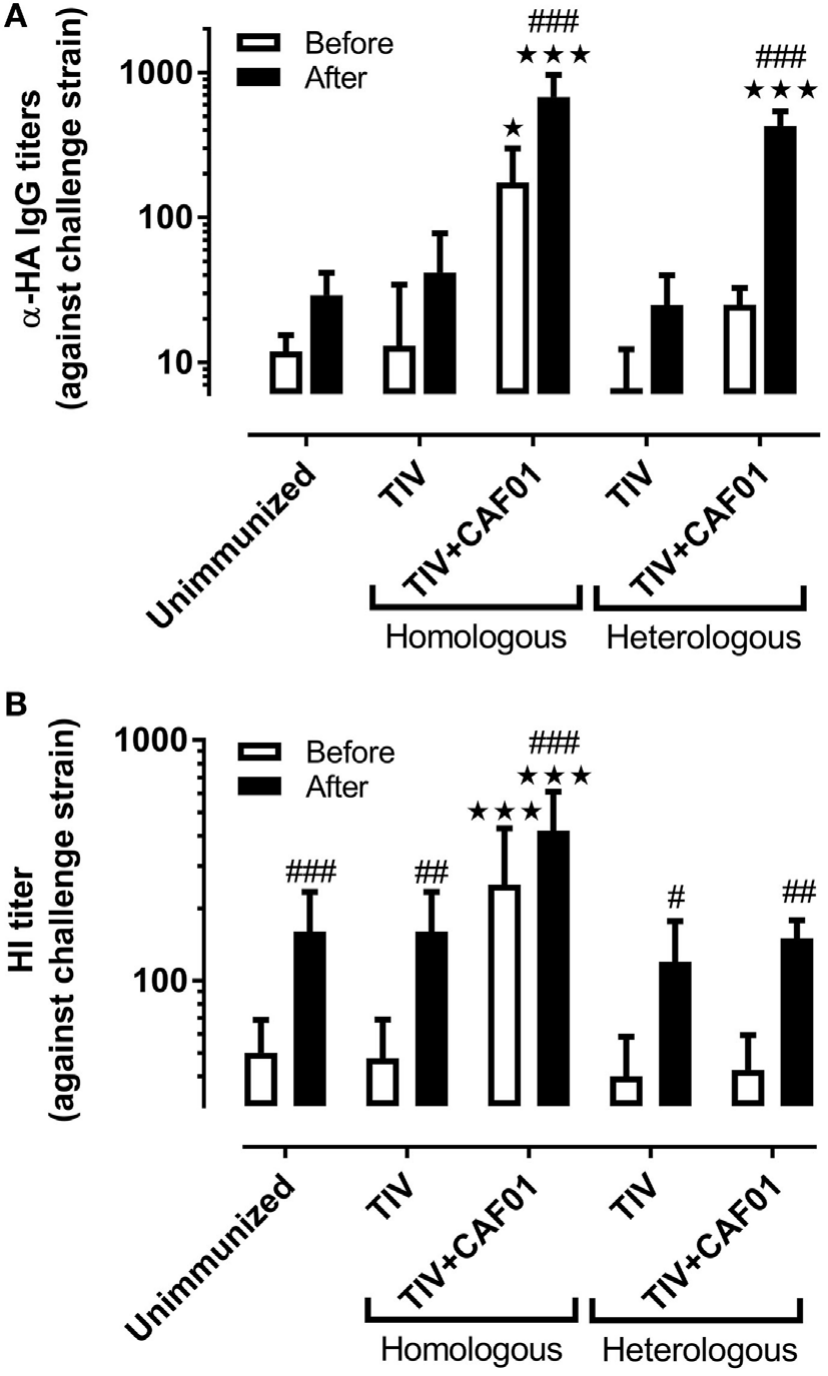
CAF01 enhances influenza-specific IgG antibody responses but not cross-reactive hemagglutination inhibition (HI) responses. Ferrets immunized two times with either the seasonal trivalent influenza split-virion (TIV)2007 or the seasonal TIV2009 vaccine with or without CAF01 adjuvant and challenged with the A/Brisbane/59/2007 influenza strain. Blood was drawn on 3 days before and 7 days after challenge. **(A)** IgG antibody titers against hemagglutinin (HA) and **(B)** HI titers against the challenge strain. Shown are mean values + SD [*n* = 8, except for **(B)**: heterologous TIV + CAF01 where *n* = 7]. Statistical analysis: two-way ANOVA based on the data shown in each graph. Sidak’s multiple comparison test was used to compare the vaccinated with the unimmunized group before (white) and after (black) infection. Star symbols show statistically significant differences compared to the unimmunized group ^★^*p* < 0.05, ^★★★^*p* < 0.001. Sidak’s multiple comparison test was used to compare the intra-group difference before and after infection and the hash symbols show statistically significant differences compared to before infection ^#^*p* < 0.05, ^##^*p* < 0.01, ^###^*p* < 0.001.

Protection against influenza is commonly associated with antibody-mediated neutralization. The ability of the different vaccines to induce IgG antibodies against the dominant surface antigen, HA, from the challenge virus was therefore investigated (Figure 2A). Only the homologous vaccine adjuvanted with CAF01 induced significant TIV-specific IgG titers before challenge. There was a slight, but insignificant, increase also for the ferrets immunized with the heterologous vaccine adjuvanted with CAF01, suggesting that some cross-reactive antibodies were induced. The regional specificity of these antibodies was not investigated but the cross-recognition could be toward conserved parts of HA, e.g., the stem region. After challenge, the specific IgG antibody response was significantly increased above the levels in unvaccinated ferrets only in those groups receiving the CAF01-adjuvanted vaccines. In addition, analysis of hemagglutination inhibition (HI) titers, which represents the correlate of protection used for humans, showed that only the CAF01-adjuvanted homologous vaccine was able to induce HI titers to the challenge strain before challenge (Figure 2B). None of the other vaccines induced HI titers above the level of the unimmunized controls at any of the investigated time points but all groups had significantly higher HI titers after challenge. Thus, the CAF01-adjuvanted heterologous vaccine was unable to induce cross-reactive protective HA-specific antibodies detectable in either of these analyses, suggesting that the partial protection obtained with heterologous vaccination was not mediated by HA inhibition (HAI).

### Clinical Symptoms

**Figure 3:**
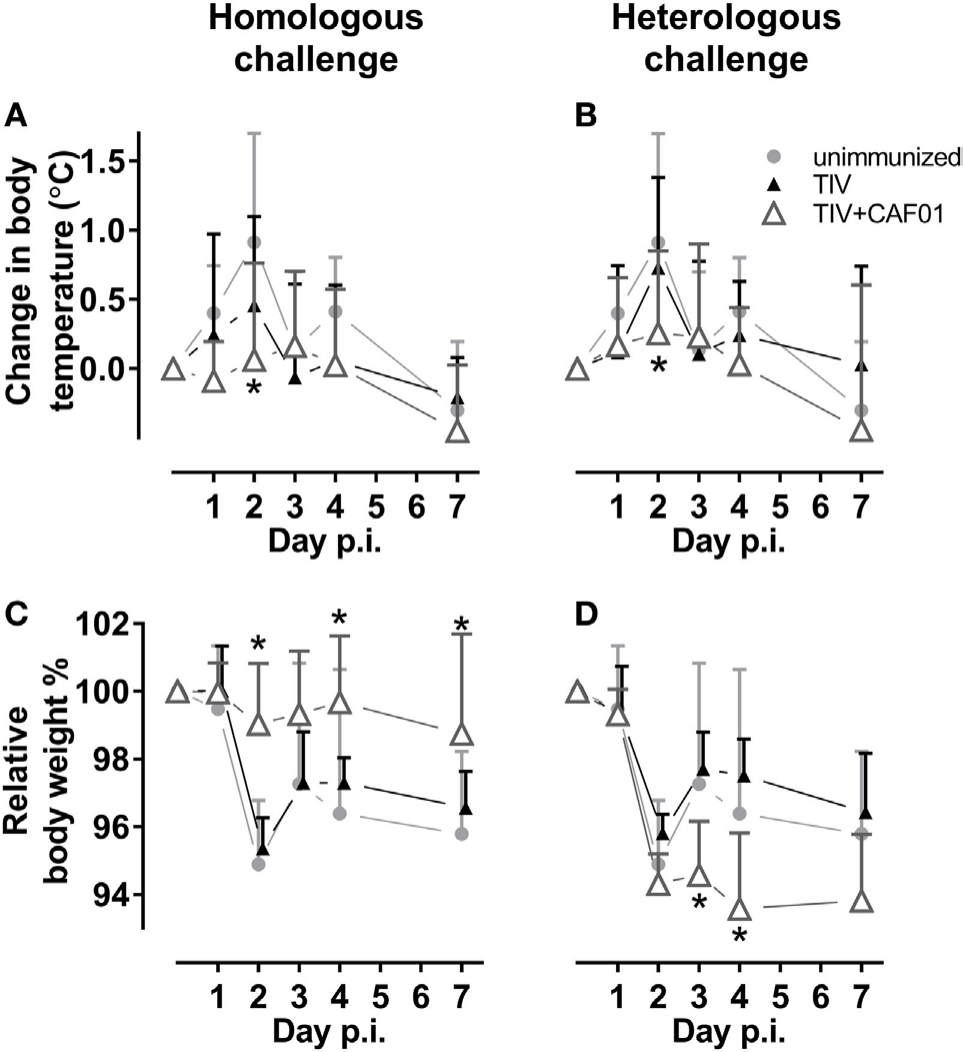
CAF01 adjuvantation reduces fever after both a homologous and a heterologous challenge. Ferrets immunized two times with either the seasonal trivalent influenza split-virion (TIV)2007 **(A,C)** or the seasonal TIV2009 **(B,D)** vaccine without adjuvant (▴) or with CAF01 adjuvant (▵). Unimmunized controls (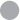). Challenge with the A/Brisbane/59/2007 influenza strain. Changes in relative body temperature **(A,B)** were determined by rectal measurements, where an average of day −1 and 0 were set to 0 and the difference was determined as that on the day of measurement minus the starting point. Relative weight **(C,D)** were determined by measurements where an average of day −1 and 0 were set to 100% and the difference was determined as day of measurement divided by the starting point. Shown are mean values + SD (*n* = 8). Statistical analysis: repeated-measures two-way ANOVA based on the data shown in each graph. Dunnett’s test was used to compare the unimmunized to the two other groups. Asterisks show statistically significant differences compared to the unimmunized group (*p* < 0.05).

We also examined if adjuvanting the vaccines could reduce clinical symptoms during influenza infection. Thus, the body temperature and body weight of the ferrets were monitored before and during challenge (Figure 3). Corresponding with the reduced viral load, only the CAF01-adjuvanted vaccines significantly protected against fever at day 2 after both homologous and heterologous challenge (Figures 3A,B). The CAF01-adjuvanted vaccine also protected the ferrets against weight loss after challenge with the homologous virus but not the heterologous vaccine (Figures 3C,D). On the contrary, the TIV + CAF01 immunized ferrets after heterologous challenge lost weight comparable to unimmunized and TIV immunized ferrets and did not gain weight again, as observed for the other groups within the 7 days the study lasted. This suggests that the TIV-CAF01 vaccine prevents systemic inflammation, whereas the weight loss could be explained by local inflammation in the airways, causing failure to thrive, and reduced food and water intake.

### Gross Pathology after Challenge

Since antibodies could not readily explain the protective effect of the CAF01-adjuvanted heterologous vaccine, we assumed that it was T-cell mediated based on the known T-cell inducing properties of CAF01. However, T-cell responses are notoriously difficult to measure in ferrets due to the lack of specific reagents of sufficient quality, so we decided to investigate if the observed protection was associated with increased influx of lymphocytes to the site of infection and/or enhanced local pathology. Thus, in a follow-up experiment with all four vaccines, ferrets were euthanized and necropsied 4 days after challenge with A/Brisbane/59/2007, as this time point is commonly associated with local pathology in ferret influenza models. Macroscopic lesions were not observed in the pharynx or in the lower respiratory tract in any of the groups.

### Histopathology after Challenge

**Figure 4:**
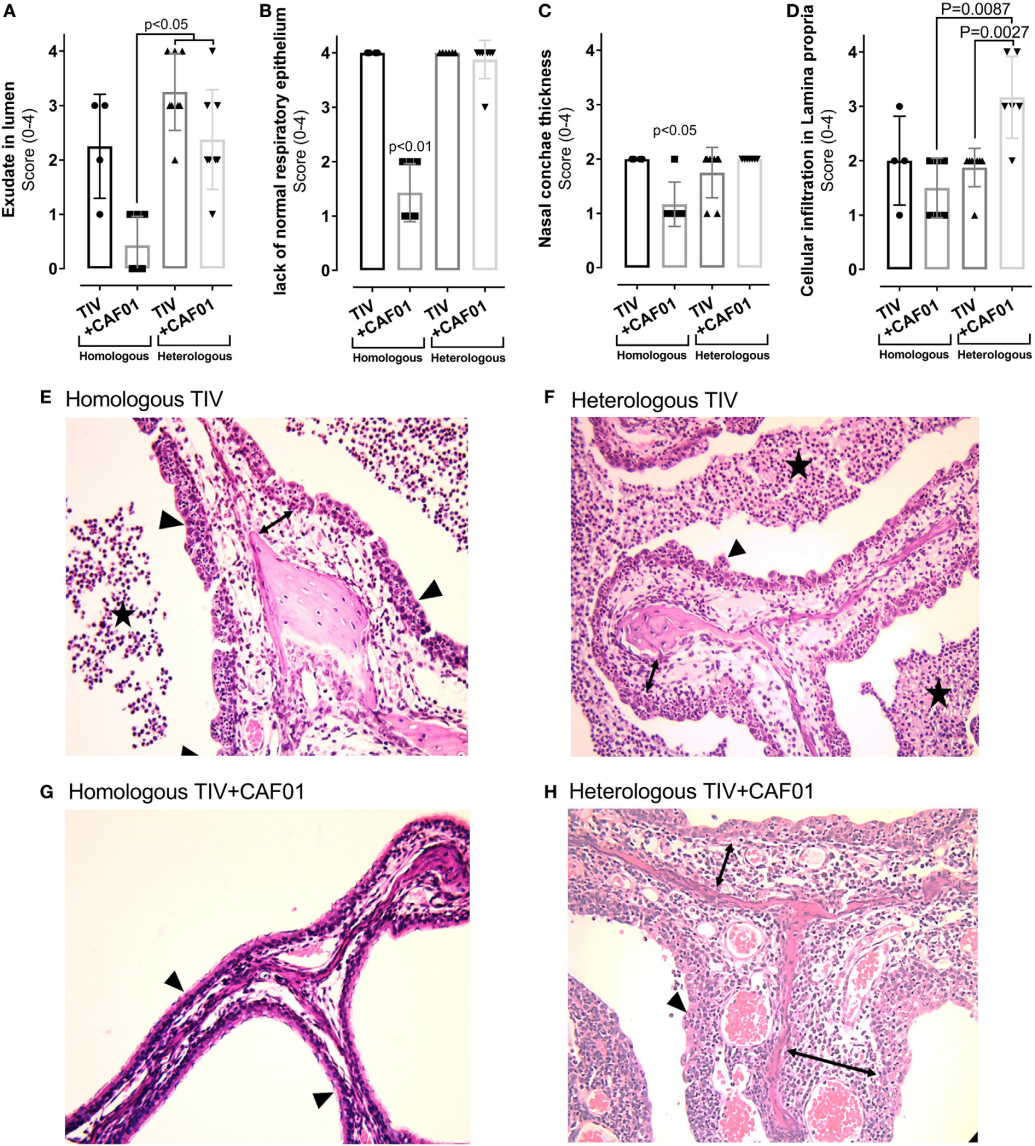
Histopathology in the nasal cavities of ferrets immunized two times with either homologous or heterologous trivalent influenza split-virion (TIV) vaccine with or without CAF01 adjuvant and euthanized 4 days after challenge with A/Brisbane/59/2007. **(A–D)** Scores of % of hematoxylin and eosin (H&E) stained sections lacking normal ciliated respiratory epithelium **(A)**, exudate in lumen of the nasal cavity **(B)**, ventral nasal concha thickness **(C)**, and degree of inflammatory cell infiltration in lamina propria **(D)**. Dots represent individual values and lines median values, *n* = 4 (homologous TIV), 6 (homologous TIV + CAF01), and 8 (heterologous TIV ± CAF01). Histological lesions observed in ferrets vaccinated with homologous TIV + CAF01 was clearly less severe compared to ferrets vaccinated with homologous TIV and heterologous TIV ± CAF01. Overall histological lesions observed in ferrets vaccinated with homologous TIV and heterologous TIV ± CAF01 were comparable; however, the degree of cellular infiltration in lamina propria was statistically significant higher in heterologous TIV + CAF01 compared to both heterologous TIV and homologous TIV + CAF01. **(E–H)** H&E-stained histological sections of nasal cavities showing unaffected thin nasal concha covered by normal ciliated respiratory epithelium from ferret vaccinated with homologous TIV + CAF01 **(G)** and clearly affected nasal cavities from ferrets vaccinated with homologous TIV **(E)**, heterologous TIV **(F)**, and heterologous TIV + CAF01 **(H)** with exudate in lumen, thickened nasal concha covered by hyperplastic epithelium lacking cilia, presence of transmigrating neutrophils and with a mixed inflammatory cell infiltration in lamina propria (most prominent in group TIV2009 + CAF01). Stars show exudate, arrowheads show epithelium, and lamina propria is marked with double-headed arrows. Bar = 500 μm. Non-parametric one-way ANOVA based on the data shown in each graph. Kruskal–Wallis test by ranks were used to compare the groups. Statistical significant difference is shown by *p*-value.

**Figure 5:**
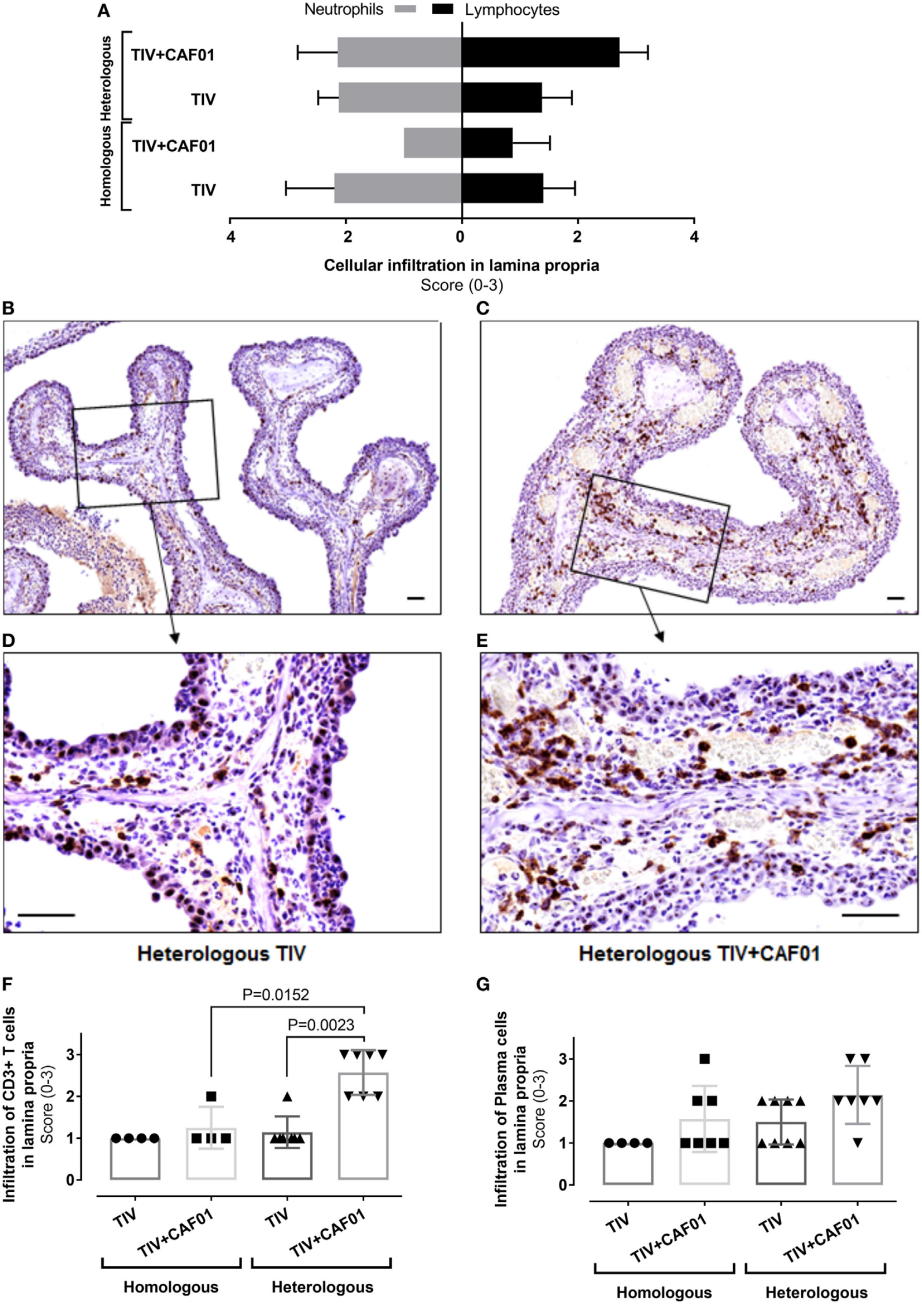
Composition of cellular infiltration to the site of infection is influenced by CAF01 adjuvantation. Ferrets immunized two times with either the homologous or heterologous trivalent influenza split-virion (TIV) vaccine with or without CAF01 adjuvant and euthanized 4 days after challenge with A/Brisbane/59/2007. **(A)** Score of number of infiltrating neutrophils’ (gray bars) and lymphocytes (black bars) in lamina propria hematoxylin and eosin (H&E)-stained sections, *n* = 4 (homologous TIV), 6 (homologous + CAF01), and 8 (heterologous TIV ± CAF01). Bars show mean value + SD. **(B–E)** Representative histological sections with immunohistochemical detection of CD3 showing brown colored CD3(+) T cells in heterologous TIV **(B,D)** and heterologous TIV + CAF01 **(C,E)**. Bars = 50 μm. **(F)** Score of number CD3(+) T cells and **(G)** plasma cells (H&E-stained section) in lamina propria. Dots represent individual values and lines median values. Statistical analysis: the Mann–Whitney two-tailed *U* test was applied to compare infiltration of cells between groups. Statistical significant difference is shown by *p*-value.

As observed from the macroscopic evaluation, histological evaluation of the pharyngeal sections revealed no differences between the four vaccination groups (data not shown). However, in the nasal cavity, there was a significantly lower degree of inflammation in the homologous TIV + CAF01 group, compared to the other vaccination groups (Figures 4 and 5).

Generally, normal ciliated respiratory epithelium was present in the majority of each examined section from ferrets in the homologous TIV + CAF01 group (Figures 4A,G), while in the other groups the nasal mucosa was completely or almost completely devoid of ciliated epithelial cells as well as goblet cells and displayed large regions with hyperplastic epithelium infiltrated by moderate to large numbers of neutrophils (Figures 4A,E,F,H; described epithelium marked with arrowheads). Regions of variable size with flattened epithelium were also present in several ferrets.

In the lumen, mild-to-severe amounts of exudate composed of neutrophils, cellular debris, and mucus was present in the majority of ferrets from the homologous TIV, heterologous TIV, and heterologous TIV + CAF01 groups, while either absent or significantly reduced in ferrets from the homologous TIV + CAF01 group (Figures 4B,E–H; exudate marked with stars).

The ventral nasal concha thickness score was statistically significant higher in the homologous TIV as well as in the heterologous TIV and TIV + CAF01 groups compared to the homologous TIV + CAF01 group, while no difference was observed between the heterologous TIV and TIV + CAF01 groups (Figure 4C). The increased thickness of the ventral nasal concha was caused by an increased inflammatory cell infiltration of the lamina propria and a mild mucosal edema (Figures 4D–H).

The degree of inflammatory cell infiltration in the lamina propria was not statistically significantly different between the homologous TIV and TIV + CAF01 groups, whereas the infiltration of inflammatory cells was statistically significantly higher in the heterologous TIV + CAF01 group than in the heterologous TIV and homologous TIV + CAF01 groups (Figure 4D). In general, all ferrets that were not protected by antibodies, i.e., those vaccinated with homologous TIV, heterologous TIV, and TIV + CAF01, experienced an increased influx of neutrophils. Furthermore, the cellular infiltration in the lamina propria was dominated by neutrophils in ferrets from the homologous and heterologous TIV groups (Figure 5A), while the cellular infiltration in the heterologous TIV + CAF01 group was dominated by substantial infiltration of mononuclear cells including lymphocytes (Figure 5A). Thus, vaccination with the CAF01-adjuvanted vaccine was associated with infiltration of mononuclear cells along with neutrophils, whereas the cell infiltration was dominated by neutrophils in those ferrets vaccinated with an unadjuvanted TIV vaccine.

Immunohistochemical (IHC) detection of CD3(+) T cells was performed on sections from the nasal cavities to investigate if heterologous vaccination was associated with a more pronounced influx of T cells compared to homologous immunization. A statistically significantly higher score of the number of infiltrating CD3(+) T cells in lamina propria was found in the heterologous TIV + CAF01 group as compared to both the homologous TIV + CAF01 and the TIV groups (Figure 5F). This is also illustrated in the representative slides of CD3 ICH stainings of histological sections of the nasal cavity from the two vaccine groups receiving heterologous challenge (Figures 5B–E). Finally, the number of plasma cells infiltrating into lamina propria after infection were scored, and no statistically significant increase was observed in the adjuvanted groups (Figure 5G).

## Discussion

It is well established that T cells play an important role in the protection against influenza infection, especially when HA-specific neutralizing antibodies are not available. Several studies have thus shown that preexisting T-cell immunity is associated with lower virus shedding and less severe illness if antibody-mediated neutralization is not possible (23–25).

The efficacy of the seasonal influenza vaccines is very low when it comes to infections with influenza strains that are not closely matched for HA, because this vaccine predominantly induces an antibody response to this antigen and T-cell immunity is not obtained (26). In this study, we therefore combined the TIV vaccines with the CAF01 adjuvant which in addition to Ab responses is known to elicit a strong T-cell response in both mice and humans. Adjuvanting the TIV vaccines with CAF01 enhanced the protective efficacy, measured as reduced viral load and fever, against both homologous and heterologous challenge. Despite the absence of detectable cross-reactive HAI antibodies, protection after heterologous challenge was not associated with an increase in local pathology when comparing to unprotected ferrets. Adjuvanting the vaccines changed the cellular recruitment to the site of infection from being dominated by neutrophilic granulocytes to lymphocytes. Increased infiltration of lymphocytes with the adjuvanted vaccines suggests that infection recruits lymphocytes that have been induced by vaccination.

This suggest that CAF01 adjuvanted the TIV vaccine increases the CMI component of the immune system, which is also supported by the histological staining of CD3+ cells in the airways after intranasal challenge, showing increased infiltration of CD3+ T cells. This rapid infiltration of T cells can lead to containment of the viral infection and faster reduction of viral load in the lungs, either by direct killing of infected cells or by mediating recruitment of innate immune cells to eliminate the infection. This correlates with observations from previous studies showing that previous heterologous infection induces a certain extent of protective immunity to novel influenza virus subtypes (27–29). In addition, Laurie et al. show that multiple infections with different influenza A strains confer better protection than a single pre-infection, suggesting that immunity to more conserved B and T cell epitopes of the influenza virus play a role for this protection (27). This was supported by the observation that primary infection by influenza strains to which the ferrets were protected by vaccination conferred reduced protection in comparison to those that were not immune to the primary infection (27, 28). Also, Bodewes et al. saw a correlation between the induction of CD3+ CD8− T cells coming from primary H3N2 virus infection and heterosubtypic protection against H5N1 virus infection (28). This role of cross-reactive T cells to mediate partial protection against a HA antigenically novel influenza virus has also been shown in non-human primates, where animals receiving a primary influenza infection had T cells coming into the to the lungs as soon as 2 days post-heterologous secondary influenza infection, in comparison to 5–7 days in non-primed animals, and that the primed animals cleared the secondary infection faster (23).

Despite the observed reduced viral load and fever symptoms following the heterologous TIV + CAF01 vaccine, we still observed weight loss in these animals. This suggest that the heterologous TIV + CAF01 vaccine prevents fever, whereas the weight loss could be explained by an increased local inflammation in the nasal cavity, as an increased influx of neutrophils and mononuclear cells was found in this group, which most likely would lead to stuffy and runny noses in those ferrets. Ferrets are obligatory nose breathers, and a stuffy nose can lead to respiratory compromise due to the ineffectiveness of mouth breathing, which again may lead to reduced food and water intake. We therefore believe that the observed weight reduction in this case was a consequence of local inflammation in the nasal cavity caused by the infection, and enhanced by the vaccine-mediated recruitment of immune cells to the airways, which on the other hand reduced the fever.

Overall, this study indicate that the combination of TIV vaccines with the TH1/TH17-inducing adjuvant CAF01 leads to increased protection against homologous influenza infection but also heterologous influenza infection despite absence of neutralization *via* HAI. Although neutralizing Ab responses to other antigens than HA can help to increase protection with the CAF01-adjuvanted vaccine, the data suggest that CMI induced infiltration of mononuclear cells play a major role, when HAI is sidelined.

## Materials and Methods

All animal experiments were approved by the Danish Animal Care and Ethics Committee and conducted in accordance with the Danish Animal Experimentation Act and the European Convention for the Protection of Vertebrate Animals used for Experimental and Other Scientific Purposes (permit no. 2012-15-2934-00503). Animals were housed in free-range area with up to 24 animals in each pen. The housing was controlled with temperature and humidity and animals were on a 12/12-h light cycle. Animals received cat food and water *ad libitum*. Animals were anesthetized with Zoletil for cats (90–120 µl dependent on their weight) when sampling and measurement of any form were performed and exsanguinated with 2-ml pentobarbital injection in the heart.

### Immunization Schedule

Outbred, 6–10 months old, female ferrets (*Mustela putorius* furo, *n* = 4–8) were randomly assigned to groups and vaccinated subcutaneous in the groin two times with 3 weeks in between, with a low dose (1/10 human dose) of either the 2007 or 2009 TIV vaccine (containing H3N2 and B, plus either A/Brisbane/59/07 or A/California/07/09 virus antigens). The TIV vaccines were used alone or mixed with the CAF01 adjuvant. A control group of unvaccinated animals received 250 µl of phosphate-buffered saline (PBS) per injection instead of the vaccine. The ferrets were housed in a class II isolation facility at the Faculty of Health Sciences, University of Copenhagen, with free access to food and water. Before vaccinations, animals were confirmed to be seronegative for circulating and applied influenza A (H1N1 and H3N2) and influenza B viruses by hemagglutination inhibition assay (HAI) and ELISA.

Eight weeks after vaccination, all animals were inoculated intranasally (i.n.) with 10^5^ TCID50 of A/Brisbane/59/2007 H1N1 produced in eggs. Thus, the challenge was homologous for one half of the vaccinated ferrets and heterologous for the other half.

Two repeat studies were performed of which one was terminated 4 days after challenge to sample tissue for histology. During challenge, nasal washes and blood samples were taken and temperatures measured daily from day 0 to day 7 post infection (p.i.). Nasal washes were performed using a pipette by application of 1 ml of PBS into the nostrils of each ferret. Nasal wash sample was collected and kept at −80°C. Blood samples were taken from the cranial vena cava. Temperature was taken by rectal measurement.

### Clinical Symptoms

Since ferrets have a large inter-individual set-point for “normal” temperature we measured the relative change in body temperature. The set point was determined as the average of the two measurements before infection in individual animals and the indicated changes were calculated as that on the day of measurement minus the set-point value.

Weight was measured during anesthesia. The starting point was determined as the average of the two measurements before infection in individual animals and this value was set to 100%. The indicated changes were calculated as that on the day of measurement divided by the starting value.

### IgG Antibodies

Detection of anti-HA antibodies in the sera from ferrets was performed by ELISA. In brief, 96-well MaxiSorp plates (Nunc, Denmark) were coated with 0.5 mg/ml of HA from either A/California/04/2009 in 15 mM Na_2_CO_3_, 35 mM NaHCO_3_ (pH 9.7), overnight at 4°C. The plates were blocked in PBS containing 2% (w/v) skimmed milk, and then the sera were added in twofold serial dilutions. On all plates an in-house positive reference serum pool was added in twofold dilutions. Specific antibodies were detected following incubation with rabbit anti-mouse IgG conjugated to horseradish peroxidase (HRP) (Zymed/Invitrogen) by addition of the TMB Plus Ready-to-use substrate (Kem-En-Tec, Taastrup, Denmark). The reaction was stopped at the optimal color development with 0.2 M H_2_SO_4_, and absorbance (OD) was read at 450 nm with wavelength correction at 620 nm to correct for optical imperfections including air bubbles in the plates. The antibody used for detection was HRP-conjugated anti IgG (Invitrogen, Frederick, MD, USA).

The relative concentration of HA-specific IgG was calculated based on the positive reference standard curve (top dilution set to 100 AU/ml). The corrected OD values were log–log transformed and the standard curve fitted manually to contain as many points as possible on a straight line (*r* > 0.99 or *n* ≥ 4). Only OD values for the dilutions of a sample within this linear interval of the reference were used to calculate the antibody titer of that sample.

Slope (*b*), intercept (*a*), and correlation (*r*) were calculated for the best fitted standard curve according to the method of least squares. According to the formula: *y* = *bx* + *a* (where *y* = OD and *x* = dilution), the relative dilution of each OD values for the samples can be calculated according to this formula: Anti-log[(((OD_sample_ − OD_background_)) − Standard-intercept(*a*))/Standard-slope(*b*)] = dilution.

The absolute concentration (titer) is then calculated by adjusting the calculated dilution with the plate dilution. This adjusted dilution is then divided into the concentration of the standard.

### Hemagglutination Inhibition

Blood was drawn from animals 3 days before and 7 days after infection with influenza and serum was prepared and stored at −20°C until use. HI titers were determined by endpoint titration. In 96-well round-bottom plates, serum was diluted twofold in 50 µl PBS and incubated with 50 µl 4 HA U influenza A/Brisbane/59/2007 for 30 min. After incubation, 50 µl 0.5% chicken RBC was added and incubated for further 60 min before hemagglutination was scored as the well with the most diluted serum that can prevent hemagglutination. Controls included were serum control (50 µl serum + 50 µl PBS + 50 µl RBC), virus control (50 µl virus + 50 µl PBS + 50 µl RBC), and erythrocyte control (100 µl PBS + 50 µl RBC). All samples were analyzed in duplicate wells.

### Quantitative Real-time Polymerase Chain Reaction (qRT-PCR)

Viral loads in nasopharyngeal swab samples collected at days 1, 2, 3, 4, and 7 were determined using qRT-PCR. The influenza M gene was absolutely quantified while the housekeeping gene, murine glyceraldehyde-3-phosphate dehydrogenase (mGAPDH), was included in the amplifications to address inter-PCR variations. The reliability of this assay was previously validated through correlation with infective titers as determined by plaque assay.

The protocol has previously been described in Andersson et al. (30). In brief, the Brilliant II qRT-PCR, 1-step kit (Agilent Technologies) was used. The following was added to each well: 100 ng purified RNA, 12.5 µl master mix, 0.5 µl of each primer (10 µM), 0.5 µl mGAPDH probe (10 µM), 0.75 µl M probe (10 µM), 0.375 µl (1:500) reference dye, and 1 µl RT/RNase block enzyme mixture diluted in Milli-Q water to a total volume of 25 µl. See Table 2 for primers and probes used.

**Table 2:** Primers and probes used for quantitative real-time polymerase chain reaction.

Primers and probes	Sequence (5′–3′)
M forward primer	AGA TGA GTC TTC TAA CCG AGG TCG
M reverse primer	TGC AAA AAC ATC TTC AAG TCT CTG
M probe	FAM-TCA GGC CCC CTC AAA GCC GA-BHQ-1
mGAPDH forward primer	CAA TGT GTC CGT CGT GGA
mGAPDH reverse primer	GAT GCC TGC TTC ACC ACC
mGAPDH probe	HEX-CGC CTG GAG AAA CCT GCC AAG TAT-BHQ-1

*Primers and probes were synthesized at TAQ Copenhagen A/S*.

*FAM, carboxyfluorescein; BHQ-1, BlackHoleQuencher-1; HEX, hexachlorofluorescein; mGAPDH, murine glyceraldehyde-3-phosphate dehydrogenase*.

Samples were run on an Mx3000P Real-time QPCR instrument (Agilent Technologies) according to the following settings: 30 min/50°C, 10 min/95°C followed by 40 cycles of 30 s/95°C, 1 min/58°C, and 30 s/72°C. All samples were run in triplicates and standard curves in duplicates. The copy number was determined with an optical density read out. The obtained results were analyzed using the Stratagene MxPro software.

### Gross Pathology and Histopathology

Four days after challenge, all ferrets were euthanized and necropsied. The ferrets heads and a segment of the pharynx were fixed in 10% neutral-buffered formalin. Following decalcification of the heads by ethylenediaminetetraacetic acid (EDTA + NaOH), a segment of the nasal cavity including the nasal concha was obtained at the level of the upper canine tooth. The tissues were routinely processed, embedded in paraffin, and sectioned. From each ferret, one section of the pharynx and one section of nasal cavity were stained with hematoxylin and eosin (H&E). In addition, one section of the nasal cavity was used for IHC detection of CD3. All processing and staining of the section was done at Nordic Biosite. All sections were examined by light microscopy by a pathologist blinded to the groups. In H&E-stained section of the nasal cavities, the following were scored: thickness of ventral nasal concha (score 1–4; 1, normal; 2, slightly increased; 3, moderately increased; 4, severely increased), lack of normal ciliated respiratory epithelium (score 1–4; 1, lacking in ≥0–25% of the section; 2, lacking in >25 to ≤50% of the section; 3, lacking in >50 to <90% of the section; 4, lacking in 90–100% of the section), the degree of inflammatory cell infiltration in lamina propria and the amount of exudate in the nasal cavity (score 0–4; 0, absent; 1, minimal; 2, mild; 3, moderate; 4, severe), the number of lymphocytes, neutrophils, and plasma cells (score 0–3; 0, absent; 1, few; 2 moderate; 3, many).

Immunohistochemical staining was performed by incubating tissue sections with monoclonal antibodies against CD3, followed by incubation with isotype-specific antibodies (DAKO). In IHC stained sections, the number of CD3(+) T-cells were scored (score 0–3; 0, absent; 1, few; 2, moderate; 3, many).

## Ethics Statement

This study was carried out in accordance with the recommendations of “Danish Animal Experimentation Act and the European Convention for the Protection of Vertebrate Animals used for Experimental and Other Scientific Purposes.” The protocol was approved by the “Danish Animal Care and Ethics Committee” (permit no. 2012-15-2934-00503).

## Author Contributions

DC, JC, KK, AT, and PA designed the study. DC, JC, KK, LI, and KE prepared laboratory work and analyzed the data. DC, KK, and PA interpreted the data and drafted the manuscript. All authors approved the final version.

## Conflict of Interest Statement

DC, KK, and PA are employed by Statens Serum Institut, a nonprofit government research facility, and holds patents related to CAF01, all rights has been assigned to Statens Serum Institut. All other authors declare that the research was conducted in the absence of any commercial or financial relationships that could be construed as a potential conflict of interest. The reviewer RR is currently coorganizing a Research Topic with one of the authors PA and confirms the absence of any other collaboration.
